# ENU mutagenesis reveals that *Notchless homolog 1* (*Drosophila*) affects *Cdkn1a* and several members of the *Wnt* pathway during murine pre-implantation development

**DOI:** 10.1186/1471-2156-13-106

**Published:** 2012-12-12

**Authors:** Amy C Lossie, Chiao-Ling Lo, Katherine M Baumgarner, Melissa J Cramer, Joseph P Garner, Monica J Justice

**Affiliations:** 1 Department of Animal Sciences, Purdue University, West Lafayette, IN, 47907, USA; 2 PULSe Interdisciplinary Life Science Program, Purdue University, West Lafayette, IN, USA; 3 Department of Medicine, Indiana University School of Medicine, Indianapolis, IN, USA; 4 Department of Comparative Medicine, Stanford University, Palo Alto, CA, USA; 5 Department of Molecular and Human Genetics, Baylor College of Medicine, Houston, TX, USA

**Keywords:** *Notchless homolog 1 (Drosophila)*, *Notch*, *Wnt*, Cdkn1a, ENU mutagenesis, Pre-implantation development, Mouse, Embryonic lethal

## Abstract

**Background:**

Our interests lie in determining the genes and genetic pathways that are important for establishing and maintaining maternal-fetal interactions during pregnancy. Mutation analysis targeted to a 34 Mb domain flanked by *Trp53* and *Wnt3* demonstrates that this region of mouse chromosome 11 contains a large number of essential genes. Two mutant alleles (*l11Jus1* and *l11Jus4*), which fall into the same complementation group, survive through implantation but fail prior to gastrulation.

**Results:**

Through a positional cloning strategy, we discovered that these homozygous mutant alleles contain non-conservative missense mutations in the *Notchless homolog 1* (*Drosophila*) (*Nle1*) gene. NLE1 is a member of the large WD40-repeat protein family, and is thought to signal via the canonical NOTCH pathway in vertebrates. However, the phenotype of the *Nle1* mutant mice is much more severe than single *Notch* receptor mutations or even in animals in which NOTCH signaling is blocked. To test the hypothesis that NLE1 functions in multiple signaling pathways during pre-implantation development, we examined expression of multiple *Notch* downstream target genes, as well as select members of the *Wnt* pathway in wild-type and mutant embryos. We did not detect altered expression of any primary members of the *Notch* pathway or in *Notch* downstream target genes. However, our data reveal that *Cdkn1a*, a NOTCH target, was upregulated in *Nle1* mutants, while several members of the *Wnt* pathway are downregulated. In addition, we found that *Nle1* mutant embryos undergo caspase-mediated apoptosis as hatched blastocysts, but not as morulae or blastocysts.

**Conclusions:**

Taken together, these results uncover potential novel functions for NLE1 in the WNT and CDKN1A pathways during embryonic development in mammals.

## Background

Mouse chromosome (*Mmu* Chr) 11 shares significant synteny conservation with regions of six different human (*Hsa*) chromosomes: 22, 7, 2, 5, 1 and 17 [1]. The largest domain of synteny conservation between mouse and human occurs on distal *Mmu* 11, which is entirely syntenic with *Hsa* 17 [2]. The gene-rich domain flanked by *Trp53* and *Wnt3* in this region of synteny conservation contains 2545 gene structures, including 1597 predicted protein-coding genes, 450 processed RNAs and 498 pseudogenes [1].

A large-scale, phenotype-driven ENU (*N*-ethyl-*N*-nitrosourea) mutagenesis screen targeted to this 34 Mb region of *Mmu* 11 demonstrated the wide functional diversity of this linkage group [2-4]. Functional analysis of 785 total pedigrees from this ENU mutagenesis screen resulted in the discovery of a variety of mutant phenotypes, including infertility, craniofacial abnormalities, neurological defects and lethality [4]. Subsequent studies detailed the embryonic lethal phenotypes of 45 mutant lines that fell into 40 complementation groups [3,4]. Resequencing efforts led to the identification of causative or putatively causative lesions in 31 genes in 17 lethal lines [1].

Although many mutations were identified in the sequencing study, the lesions in the *l11Jus1* and *l11Jus4* complementation group have yet to be identified. These two alleles survive through implantation but arrest prior to embryonic day (E) 6.5 [3,4]. Our interests lie in determining the genes and genetic pathways that are important for establishing and maintaining maternal-fetal interactions during pregnancy. Since these two mutants fail during this critical window, we undertook a positional cloning strategy to identify the causative mutations in this complementation group. Here, we present evidence that both mutant alleles have non-conservative missense mutations in the *Notchless homolog 1* (*Drosophila*) gene, *Nle1*. Moreover, targeted disruption of *Nle1* in mice [5] results in an embryonic lethal phenotype that is remarkably similar to *l11Jus1* and *l11Jus4*, providing further supporting evidence that *Nle1* is disrupted in both mutant alleles.

NLE1, which is a member of the WD40 repeat protein family, was first identified as a suppressor of the *notchoid* phenotype in *Drosophila*[6], and has been implicated in both positive and negative regulation of NOTCH signaling, depending upon developmental stage and species [5,6]. Studies in *Drosophila* and *Xenopus* demonstrate that NLE1 signals via the canonical NOTCH pathway [5,6]. In invertebrates and lower vertebrates, the NOTCH pathway is critical for directing cell fate prior to gastrulation, and also plays important, but varied roles in germ layer boundary formation. At the 4-cell stage in *C. elegans*, NOTCH signaling dictates an ectodermal cell fate in ABp daughter cells by repressing expression of *TBX-37* and *TBX-38*[7]. In sea urchins, the NOTCH pathway impacts the development and differentiation of the secondary mesenchymal cells, which are fated to produce mesodermal cells [8,9]. In contrast, in *X. laevis*, induction of NOTCH signaling leads to an increase in endoderm-specific and a decrease in mesoderm-specific markers, while suppression of NOTCH signaling has the opposite consequence [10].

The role of NOTCH signaling during the earliest stages of mammalian development is much less clear. Several lines of evidence demonstrate that NOTCH signaling is dispensable for gastrulation in mice. Single gene and compound knockout studies of the *Notch* receptors and ligands results in either viable animals or embryonic lethality at mid-gestation [11-21]. Similarly, deletion of genes that block NOTCH signaling, such as *Pofut1* and members of the γ-secretase complex, leads to embryonic failure after gastrulation and midline formation. POFUT1 adds O-fucose molecules to NOTCH receptors prior to their translocation to the cell surface, while Presenilin 1 and 2 are members of the γ-secretase complex [22,23]. This complex cleaves NOTCH at the cell membrane, releasing the NOTCH intracellular domain (NICD) into the cytoplasm. The NICD translocates to the nucleus and binds to RBPJ, thereby modulating transcription of downstream target genes.

Deletion of *Pofut1*, which effectively blocks NOTCH signaling through inhibition of post-translational modifications to NOTCH receptors [24], leads to embryonic lethality at E9.5 [25,26]. Targeted disruption of *Presenilin 2* in a *Presenilin 1* null background leads to embryonic lethality at E9.5. Compound mutants exhibit cardiac, somite and neurological phenotypes [27]. Finally, deletion of the co-repressor, *Rbpj*, causes somitogenesis defects, placental abnormalities and marked growth delay [28,29]. These studies demonstrate that unlike lower vertebrates and invertebrates, and despite the fact that *Notch* receptors and ligands are expressed prior to and during gastrulation [30], NOTCH signaling is dispensable prior to gastrulation in mice.

Since *Nle1*^*l11Jus1*^ and *Nle1*^*l11Jus4*^ mutants have more severe phenotypes than mutations that disrupt NOTCH signaling in mice, we hypothesized that NLE1 interacts with NOTCH and other signaling pathways during pre-implantation development. To address this hypothesis, we conducted targeted gene expression studies in homozygous mutant embryos. Surprisingly, and in contrast to studies in *Xenopus* and *Drosophila*, our data indicate that canonical NOTCH signaling is not disrupted in *Nle1* mutant embryos; instead, we discovered that *Cdkn1a* was upregulated, while several members of the *Wnt* cascade were downregulated in homozygous mutant embryos. These results highlight the differences in NOTCH signaling between mammals (where canonical NOTCH signaling is dispensable for gastrulation) and other species (where NOTCH signaling is required for gastrulation) and indicate that NLE1 could play divergent roles in development that depend upon other signal transduction cascades.

## Methods

### Mouse strains, meiotic mapping and generation of mutant embryos

**Figure 1:**
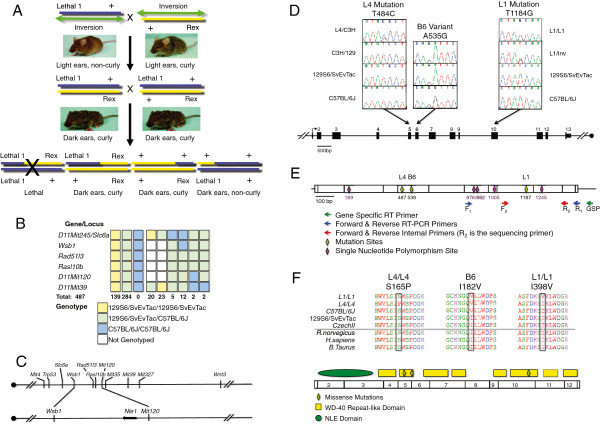
**Positional Cloning of *l11Jus1 *and *l11Jus4*. A**. *Exclusion Mapping Breeding Scheme.* Blue line: ENU-mutated allele (*l11Jus1*; C57BL/6J); Green line with the double arrow: 34 Mb inversion (*In*(*11Trp53;11Wnt3*)*8Brd*; 129S6/SvEvTac); yellow line: dominant *Rex* allele (129S6/SvEvTac), which confers a curly coat. Plus sign: wild type locus. All animals carrying two copies of the *l11Jus1* allele will die *in utero*. Representative phenotypes and crossover events are depicted in the F_2_ generation. **B**. *Mapping the* Nle1 *mutation by haplotype analysis in the F*_*2*_*generation.* Each box represents a locus within *Mmu* 11. Yellow boxes are homozygous 129S6/SvEvTac genotypes; green boxes are heterozygous for 129S6/SvEvTac and C57BL/6J genotypes; while blue boxes indicate homozygous C57BL/6J genotypes. *Wsb1* &*D11Mit120* define the boundaries of the critical region. **C**. *Physical map of the non-recombinant interval. Nle1* lies ~700 kb centromeric to *D11Mit120*. **D**. *Mutation analysis.* Arrows indicate the location of each mutation. Stacked chromatographs show the sequence for each strain. **E**. *The* Nle1 *cDNA and genotyping primers.* The full-length, spliced mRNA product is shown with exons represented by empty boxes. Primer locations are depicted by arrows. The 5’ and 3’UTRs are represented by a thin line on the mRNA transcript. **F**. *NLE1 protein structure.* Green oval: NLE specific domain; yellow boxes: WD40 Repeat-like domains; green diamonds: mutation sites.

The *l11Jus1* and *l11Jus4* mutants were induced by ENU mutagenesis on a C57BL/6J background [3,4], and maintained in *trans* using a balancer chromosome (*In*(*11Trp53;11Wnt3*)*8Brd*) harboring a 34 Mb inversion between *Trp53* and *Wnt3* that expresses the agouti protein under the *Keratin 14* promoter (Figure 1A) [31]. The inversion animals were on a 129S6/SvEvTac background. Animals carrying one copy of the balancer chromosome have light ears and tails due to ectopic agouti expression that reduces pigment [3,4,31,32]. The *l11Jus1* line has been continually maintained in our colony. The *l11Jus4* line was resuscitated from cryopreserved spermatozoa of an *l11Jus4/In*(*11Trp53;11Wnt3*)*8Brd* male with a C3H/HeJ female (http://www.MMRRC.org, MMRRC:000074-UCD). Pups were genotyped at weaning and *l11Jus4/*C3H/HeJ males were backcrossed to *In*(*11Trp53;11Wnt3*)*8Brd*/*Rex* females on a 129S6/SvEvTac genetic background. We genotyped the progeny of both lines at least every 10 generations by microsatellite analysis or direct sequencing of the mutations to ensure that we maintained the mutations.

To generate recombinant animals for meiotic mapping it is necessary to remove the balancer chromosome. Animals heterozygous for the *l11Jus1* mutation (*l11Jus1/In*(*11Trp53;11Wnt3*)*8Brd*) were mated to animals carrying one copy of the inversion and the dominant curly coat marker, *Rex* (*In*(*11Trp53;11Wnt3*)*8Brd*/*Rex*). We selected animals with a curly coat (i.e. inherited the *Rex* allele) and dark ears and tail (i.e. inherited the *l11Jus1* mutation) for meiotic mapping. We intercrossed F_1_ animals to generate recombinant F_2_ animals, which were genotyped at several microsatellite markers (*D11Mit4*, *219*, *245*, *120*, *39*, *327*, and *32*) and single nucleotide variances (SNVs; *Slc6a4*, *Wsb1*, *Rad51l3* and *Rasl10b*) along the 34 Mb interval (Figure 1B). Primers and PCR conditions are available upon request.

For the *Notch* PCR array studies, qRT-PCR analysis and caspase 3 detection, we outcrossed heterozygous males to 129S6/SvEvTac females (Taconic, Hudson, New York) to eliminate genetic interactions with *Wnt3*. Heterozygotes, which had dark ears and tails were mated, generating F_2_ blastocysts for analysis. *Notch* PCR array studies were conducted on N5F_2_ embryos (*Nle1*^*l11Jus1*^). qRT-PCR studies were performed on N14F_2_ and N15F_2_ (*Nle1*^*l11Jus1*^) and N4F_2_ (*Nle1*^*l11Jus4*^) embryos dissected at E3.5. Caspase detection assays were carried out on N15F_2_ (*Nle1*^*l11Jus1*^), as well as N14F_2_ or N15F_2_ (*Nle1*^*l11Jus4*^) embryos. All mouse studies were conducted in facilities approved by the American Association for the Accreditation of Laboratory Animals with the approval of the Baylor College of Medicine Animal Care and Use Committee or the Purdue University Animal Care and Use Committee.

### Embryo analysis

To determine time of death and perform phenotypic studies, we examined embryos after timed matings, with the day of the vaginal plug designated E0.5. We genotyped each one as described [3,4]. DNA was isolated by incubating whole embryos (E6.5 to E9.5) in 1 X PCR buffer (Life Technologies, Grand Island, NY) and 0.08 mg/ml Proteinase K (Life Technologies, Grand Island, NY) at 55°C for 2–3 hours. Proteinase K was inactivated by either heating to 95°C for 10 min or by phenol:chloroform:Isoamyl alcohol extraction followed by ethanol precipitation. Alternatively, embryos were incubated in 25 to 50 μl of 25 mM NaOH, 0.2 mM EDTA for 60 min at 95°C. Genomic DNA was neutralized by the addition of an equal amount of 40 mM Tris–HCl, pH 8.0 and stored at −20°C. *D11Mit327* was used to genotype the embryos in a 25 μl PCR reaction under the following conditions: 1 X PCR buffer, 1.5 mM MgCl_2_, 0.2 mM dNTPs, 250 pmoles of each primer and 0.625 U of *Taq* Polymerase (Life Technologies, Grand Island, NY). After an initial denaturing step at 95°C for 5 min, *D11Mit327* was amplified with the following cycling parameters: 30 cycles of 94°C for 30 s, 60°C for 30 s followed by 72°C for 30 s, with a final 5 min incubation at 72°C. Products were size fractionated on 5% Metaphor (Cambrex, Bio Science, Rockland, ME), 0.5 X TBE gels.

### Histology

Deciduas were dissected at E6.5–E8.5. Implantation sites were fixed for 3 hours in Bouin’s fixative, embedded in paraffin, sectioned in 5–7 μm slices and stained in hematoxylin and eosin as described [33]. Stained sections were analyzed under light microscopy.

### Candidate gene interrogation

Exons of candidate genes were bidirectionally sequenced directly from PCR amplicons using the Big Dye® Teminator v3.1 (Life Technologies, Grand Island, NY) sequencing mix. Each amplicon contained at least one exon, plus ≥ 200 bp of flanking sequence. For the *l11Jus1* mutation, genomic DNAs from 129S6/SvEvTac, C57BL/6J and *l11Jus1/In*(*11Trp53;11Wnt3*)*8Brd* mice were sequenced as controls. For the *l11Jus4* mutation, genomic DNAs from 129S6/SvEvTac, C57BL/6J, *In*(*11Trp53;11Wnt3*)*8Brd*/C3H, and *l11Jus4/C3H, and l11Jus4/In*(*11Trp53;11Wnt3*)*8Brd* mice were sequenced as controls. Sequence data was analyzed (Sequencher; Gene Codes, Ann Arbor, MI) to identify mutations on the *l11Jus1* and *l11Jus4* alleles, as well as any additional sequence variants.

### Notch pathway expression of l11Jus1 mutants at E3.5

We analyzed *Notch* pathway gene expression in homozygous mutant and homozygous wild-type blastocysts using the SAB PCR Arrays (SABiosciences/Qiagen, Frederick, MD). Embryos were washed in EmbryoMax© M2 media (M2; EMD Milllipore, Billerica, MA) and transferred into 100 μl of RNAqueous lysis buffer (RNAqueous-Micro Kit, Life Technologies, Grand Island, NY). After vortexing, we snap-froze each tube in liquid nitrogen and stored each sample at −80°. Total RNA was isolated following manufacturer’s instructions, eluting with 20 μl of nuclease-free water. We used 5 μl of RNA to generate cDNA for genotyping in a half reaction of SuperScript One-Step RT-PCR with Platinum Taq (Life Technologies, Grand Island, NY) with gene specific primers, oligo dT, and PCR primers (Table 1). We used nested PCR for sequencing (Figure 1E). We collected 45 mutant and 45 wild-type E3.5 embryos, and split embryos with the same genotype into 3 pools (i.e. biological replicates); each pool consisted of morulae, half-blastocysts, full blastocysts and hatched blastocysts. We then performed a linear amplification step on each pool using the RT^2^ Nano PreAMP cDNA synthesis kit (SABioscience, Frederick, MD). Each biological replicate was subdivided into 3 technical replicates. Data from each PCR plate were analyzed using an iCycler Real Time PCR detection system (Bio-Rad, Hercules, CA).

**Table 1:** Primers used for this study

	**Forward sequence**	**Reverse sequence**	**Tm**	**Size**
***Nle1 *sequencing primers**				
*Nle1E1/2*	CTTGACTCCTCCGAACACGAG	AAACACAGCCTGTCTGTAGGTGAG	62	500
*Nle1E3*	GATTAAATTTGTCGCATGGTGGTA	GTCTGTTACTTGCAACGTGAGTCC	62	475
*Nle1E4*	TATTTCTCCTCAGGGAATGGAGAG	CCACACTCAGTCCAGTATCTGCTT	62	377
*Nle1E5/6*	CTGTGTTCTCCCTCACCTCTCC	ATAGTAGGCCAAGCCGTTGCT	62	557
*Nle1E7*	ACAGCCTTGCTCTGCTGTTAGAA	GGACCAGCTGGACTCTTGGTATAA	62	440
*Nle1E8/9*	TTCCTGATTCTTGCCTTATGTCAC	AACCCTAACTAAGACAACCAAGAACAA	62	544
*Nle1E10*	TGGAGTTGCATGTAAGCTTGTGT	GTCACTAGCCCTAAAGATGCCATT	62	488
*Nle1E11/12*	CCGGCCCAGGTACCTAGCTT	ACCTACAGGTTCTCCCAGAGTCTCC	62	498
*Nle1E13*	ACTTGATACTTGGCAGTAGGCACA	CTCCTGCTATCCAGTGCAAGG	62	570
***Nle1 *genotyping primers**				
*Nle1* GSP	GCTGTAATGTCCTGACTGT		60	637
cDNA 1	CTGTGTCGTACTCTTCAAGGTCAT	CTGTGGAGTCATCTTCTCCATATC		
cDNA 2	TCAGACGACTTCACCTTATTCCTG	CAGTCAACAGCATATACCTCATCG	62	351
*Nle1* DNA	TCTCCTTCAGCTCCTTCACTGT	TCCAATGGTGGAGTATAGGGTATAA	60	341
**cDNA amplification primers**				
1^st^ PCR	ATATGGATCCGGCGCGCCGTCGACT_24_	ATATCTCGAGGGCGCGCCGGATCCT_24_	67	
2^nd^ PCR	(NH2)ATATGGATCCGGCGCGCCGTCGACT_24_	(NH2)ATATCTCGAGGGCGCGCCGGATCCT_24_	67	
*Eed*	GTGTGACATTTGGTACATGAGGTT	ACATTTATGATGGGTCAGTGTTGT	60	148
**TaqMan gene expression assay**				
	Assay ID	Amplicon Size (bp)		
*Trp53*	Mm01731290_g1	119		
*Cdkn1a*	Mm04205640_g1	80		
*Gapdh*	Mm99999915_g1	107		

### PCR array data analysis for gene expression

Cycle threshold (Ct) values were calculated for all data obtained from 18 PCR plates. We calculated the optimal threshold values based on the value for each plate by selecting the auto calculate threshold position and the PCR base-line subtracted analysis mode from the iCycler Data Analysis Software (Bio-Rad, Hercules, CA). The highest threshold position was 1415 PCR base-line subtracted relative fluorescence units (RFU). We re-analyzed each plate by entering 1415 as the user defined threshold position. Therefore, we were able to compare replicates across multiple plates using Ct values generated from the common threshold position.

We used the SABiosciences RT2 Profiler Data Analysis Software to determine gene expression profiles (http://www.sabiosciences.com/pcr/arrayanalysis.php). This software calculated fold regulation values for each gene using the relative quantification 2^-ΔΔCt^ method [34]. Each plate met the quality assurance criteria listed by the manufacturer for genomic DNA contamination, reverse transcription inhibition, and PCR cycling conditions. ΔCt values were normalized using the mean values of three housekeeping genes: *Gusb*, *Hsp90ab1*, and *Actb*. All wells with a Ct value above 29.5 cycles were excluded from the analysis. This left 65 transcripts for analysis.

### Caspase 3 detection

Active caspase were detected based on a fluorescent inhibitor of caspase (FLICA) approach [35,36]. Zona-free embryos were placed on slides and incubated with FLICA caspase 3 reagent (Image-iT™ LIVE Red caspase-3 and −7 Detection Kit, Life Technologies, Grand Island, NY) in M2 medium at 37°C for one hour. FLICA was removed and the embryos were washed with M2 media, counterstained with Hoechst dye for 3 minutes and washed with buffer provided by the manufacturer. Embryos were fixed in 1% PFA for 10 min and mounted on cover slides. Each embryo was imaged with Zeiss LSM510 microscope (20X objective) and the images were pseudo-colored using Adobe Photoshop (Adobe Systems, Inc., San Jose, CA). To genotype, we collected each embryo in 10 μl of 100 μg/ml Proteinase K solution, incubated the embryos at 55°C for 10 minutes, and then heat inactivated at 95°C for 5 minutes. We used these lysates to genotype each embryo by two rounds of PCR using primers that flanked an insertion/deletion in exon 8*.*

### Quantative RT-PCR (qRT-PCR) by TaqMan

RNA isolated from embryos separated by genotype (mutant vs. wild-type) and stage (morula, full blastocyst and hatched blastocyst) was reverse transcribed individually following the protocol by Tang and Colleagues [37]. Following a 1:1 addition of 100% ethanol, RNA was concentrated with a SpeedVac for 15 mins, resuspended in 4.5 μl lysis buffer and reverse transcribed. We performed a two-step linear amplification process using barcoded primers as described [37]. Products from the first and second rounds were purified using Zymo DNA concentration kits (Zymo Research, Irvine, CA) and eluted in 30 μl of 1 X T_10_E_0.1_. *Eed* expression was used to check the cDNA quality following the first linear amplification step (primers listed in Table 1). cDNA was quantified using a Bio-Rad SmartSpec™ Plus Spectrophotometer. Ten ng of cDNA was used as a template for qRT-PCR in combination with TaqMan® Gene Expression Master Mix (PN#4369514; Life Technologies, Grand Island, NY) and Taqman Gene-specific probes (Life Technologies, Grand Island, NY) on a Prism 7000HT Sequence Detection System (Life Technologies, Grand Island, NY). We assayed a minimum of three biological replicates for each group. Cycling reactions were performed in duplicate or triplicate. The relative expression of each gene was calculated based on the ΔΔCt value, where the results were normalized to the average Ct value of *Gapdh*. Samples that failed to generate a signal above threshold at the end of the reaction were given a Ct value of 40.

### Statistical methods

*SAB PCR Array Study*. These data can be thought of as a complex nested block design: Plate is nested within Pool and Pool is nested within Genotype (Plate and Pool are both blocks). Transcript is nested within biological Role (each transcript was assigned one biological Role), and transcript and role are crossed with each level of nesting (i.e. each transcript is measured on each plate). The data were analyzed in a GLM, blocked by Plate nested within Pool, and Pool nested within Genotype. Transcript was nested within Role; and Role and Transcript crossed with the blocking factors Plate and Pool, and with the experimental factor Genotype. Each Plate and Pool acted as its own control. The relationship of Plates as technical replicates from the same Pools is recognized. Transcript describes the overall expression profile, while Role describes the overall Functional Profile, and their interactions with Genotype test (respectively) whether particular Transcripts differ from the average for the Role between Genotypes, and whether particular Roles differ as a whole between Genotypes. We partitioned out between-plate error and used this as the error term for analyses for two reasons: 1) the plate reader software controls within plate error and 2) the use of between-plate error is conceptually equivalent to (the source of error in a traditional ANOVA approach testing each gene independently. By using ΔCt values, the analysis directly calculates ΔΔCt.

*qRT-PCR study by TaqMan analysis.* We adopted a similar GLM approach to individually test and calculate the ΔΔCt values from *Cdkn1a* and *Trp53* gene expression studies. Since we used the –ΔCt for each individual as raw data, genotype interactions figures and tests a ΔΔCt value. We also tested for common changes in gene expression in different stages of pre-implantation development and used the full pairwise comparisons table to generate the individual ΔΔCt values and standard errors.

## Results

### *Phenotypic analysis of* l11Jus1 *and* l11Jus4

**Figure 2:**
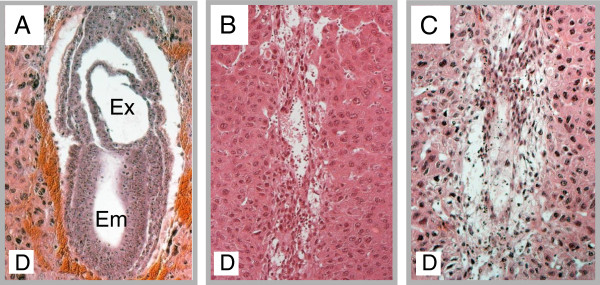
**Mutant Phenotypes.** H&E stained sections at E7.5 **A**. Wild type implantation site **B**. *l11Jus1* implantation site. **C**. *l11Jus4* implantation site. embryo (Em), extra-embryonic region (Ex), and maternal decidua (**D**).

We screened a cohort of 59 lethal mutants (45 of which were embryonic lethal) that were generated by ENU mutagenesis [3], and identified an allelic series of two mutants (*l11Jus1* and *l11Jus4*) mapping to mouse chromosome 11 that failed to gastrulate. Histological sections performed at embryonic day (E) 7.5 show completely resorbing implantation sites compared to control littermates (Figure 2). In contrast, animals inheriting two copies of the 34 Mb inversion, *In*(*11Trp53;11Wnt3*)*8Brd*, are homozygous mutant for *Wnt3* and display a distinct, much less severe phenotype during the gastrulation stage (Table 2) [3,4]. Complementation studies revealed that the phenotype of the *l11Jus1/l11Jus4* double heterozygotes is identical to either single homozygous mutant (data not shown), thereby placing *l11Jus1* and *l11Jus4* in the same complementation group.

**Table 2:** Time of death for Nle1 mutants

**A. Time of death for *l11Jus1 *Mutants**											
		**Abnormal**				**Normal**					
**Day**	**Resorbed**	**No DNA**	**L1/L1**	**L1/Inv**	**Inv/Inv**	**No DNA**	**L1/L1**	**L1/Inv**	**Inv/Inv**	**Lost**	**Total**
**3.5**	0	2	2	4	3	40	32	58	23	1	165
**6.5**	28	0	0	0	18	0	0	42	0	10	98
**7.5**	3	0	0	0	1	0	0	5	0	3	12
**8.5**	3	0	0	2	3	0	0	1	0	4	13
**9.5**	4	0	0	1	1	0	0	4	0	0	10
**Total**	38	2	2	7	26	40	32	110	23	18	298
**B. Time of death for *l11Jus4 *Mutants**											
		**Abnormal**				**Normal**					
**Day**	**Resorbed**	**No DNA**	**L4/L4**	**L4/Inv**	**Inv/Inv**	**No DNA**	**L4/L4**	**L4/Inv**	**Inv/Inv**	**Lost**	**Total**
**3.5**	0	2	0	8	3	6	28	86	27	1	161
**6.5**	16	0	0	4	9	0	0	19	0	0	48
**7.5**	7	0	0	0	4	0	0	19	0	0	30
**8.5**	13	0	0	5	5	0	0	11	0	0	34
**9.5**	10	0	0	0	1	3	0	1	0	0	15
**Total**	46	2	0	17	22	9	28	136	27	1	288

*Penetrance of* l11Jus1 (L1) (Table 2A): We genotyped a total of 34 *l11Jus1* (*L1/L1*) homozygotes (32 normal and 2 abnormal blastocysts), 117 heterozygotes (*L1/Inv*) and 49 animals homozygous for the inversion (*Inv/Inv*). We failed to genotype 98 embryos due to lack of DNA from normal (n = 40) and abnormal (n = 2) embryos; resorption sites (n = 38) and lost embryos (n = 18) accounted for the remainder of non-genotyped embryos. At E6.5, we detected 0 homozygous mutant embryos out of 62 total embryos. *X*^2^ analysis indicates that these numbers are statistically significant, with p < 0.0001 (Table 3). At the blastocyst stage (E3.5), we detected normal Mendelian ratios, indicating that the time of death occurs between E3.5 and E6.5.

**Table 3:** ***X***^**2**^**Analysis of selected timed matings**

**Genotype**	**Observed**	**Expected**	**Stage**	**Total embryos**	**P value**
***l11Jus1/l11Jus1***	34	50	All	200	0.018
***l11Jus1/In(11Trp53;11Wnt3)8Brd***	117	100			
***In(11Trp53;11Wnt3)8Brd/In(11Trp53;11Wnt3)8Brd***	49	50			
***l11Jus1/l11Jus1***	34	30.5	E3.5	122	0.582
***l11Jus1/In(11Trp53;11Wnt3)8Brd***	62	61			
***In(11Trp53;11Wnt3)8Brd/In(11Trp53;11Wnt3)8Brd***	26	30.5			
***l11Jus1/l11Jus1***	0	15	E6.5	60	3.71703E-05
***l11Jus1/In(11Trp53;11Wnt3)8Brd***	42	30			
***In(11Trp53;11Wnt3)8Brd/In(11Trp53;11Wnt3)8Brd***	18	15			
***l11Jus4/l11Jus4***	28	57.5	All	230	5.17678E-07
***l11Jus4/In(11Trp53;11Wnt3)8Brd***	153	115			
***In(11Trp53;11Wnt3)8Brd/In(11Trp53;11Wnt3)8Brd***	49	57.5			
***l11Jus4/l11Jus4***	28	38	E3.5	152	0.014
***l11Jus4/In(11Trp53;11Wnt3)8Brd***	94	76			
***In(11Trp53;11Wnt3)8Brd/In(11Trp53;11Wnt3)8Brd***	30	38			
***l11Jus4/l11Jus4***	0	8	E6.5	32	0.004
***l11Jus4/In(11Trp53;11Wnt3)8Brd***	23	16			
***In(11Trp53;11Wnt3)8Brd/In(11Trp53;11Wnt3)8Brd***	9	8			

*Penetrance of* l11Jus4 (L4) (Table 2B). We genotyped a total of 28 *l11Jus4* (*L4/L4*) homozygotes (all normal), 153 heterozygotes (*L4/Inv*) and 49 animals homozygous for the inversion (*Inv/Inv*). We failed to genotype 58 embryos due to lack of DNA from normal (n = 9) and abnormal (n = 2) embryos; resorption sites (n = 46) and lost embryos (n = 1) accounted for the remainder of non-genotyped embryos. At E6.5, we detected 0 homozygous mutant embryos out of 32 total embryos. *X*^2^ analysis indicates that these numbers are statistically significant, with a p-value of 0.004 (Table 3). At the blastocyst stage (E3.5), we detected all genotypes, but saw an unexpectedly high number of heterozygotes (p = 0.014). Together, these data indicate that *l11Jus1* homozygotes and *l11Jus4* homozygotes both die *in utero* prior to E6.5.

### *Positional cloning of* l11Jus1 *and* l11Jus4

Since *L1* and *L4* homozygotes failed at the implantation stage, meiotic mapping would be difficult, at best, using traditional methods that rely on haplotype analysis in phenotypically mutant animals. To circumvent this obstacle, we narrowed the critical interval by exclusion mapping (Figure 1A). Exclusion mapping involves haplotype analysis of all progeny at weaning for several markers across the candidate interval (i.e. from *Trp53* to *Wnt3* on Mmu 11). Since homozygous mutants are embryonic lethal, any marker that is homozygous for the mutant allele (i.e. C57BL/6J) will effectively ‘exclude’ this marker from the candidate interval. Parents were heterozygous for the *l11Jus1* mutation and for the dominant coat color marker, *Rex*. Throughout the 34 Mb critical interval, *l11Jus1* is on a C57BL/6J background, while *Rex* is on a 129S6/SvEvTac background. Since the balancer chromosome is not present in the F_1_ generation, it is possible to obtain animals that have recombination events on one or both parental alleles. These recombination events were visualized by haplotype analysis in the F_2_ generation (Figure 1B). We genotyped 487 progeny (974 individual meiotic events), and narrowed the critical region to a 4.4 Mb domain flanked by *Wsb1* and *D11Mit120* (Figure 1B, C).

Of the 75 genes in this interval, 16 top candidates were selected based on microarray expression data and mutant phenotype. We sequenced 8 of these genes in the process of identifying the *l11Jus1* and *l11Jus4* mutations: *adaptor-related protein complex 2, beta1 subunit* (*Ap2b1*); *chaperonin containing Tcp1, subunit 6b (zeta)* (*Cct6b*); *suppressor of zest 12 homolog (Suz12*); *fringe isoform 1* (*Rffl*); *ecotropic viral integration site 2a* (*Evi2a*); *proteasome (prosome, macropain) 26S subunit non-ATPase 11* (*Psmd11*); *TAF15 RNA polymerase II, TATA box binding protein (TBP)-associated factor* (*Taf15*); and *Notchless homolog 1* (*Drosophila*) (*Nle1*).

We found no non-synonymous mutations in our first 7 candidates (*Ap2b1*, *Cct6b, Suz12, Rffl, Evi2a, Psmd11 or Taf15*) [38]*.* However, we identified a T 1184 G transversion (I 395 S missense mutation) in *l11Jus1* heterozygotes in exon 10 of *Nle1* (Figure 1D). This non-conservative substitution replaces an aliphatic, hydrophobic amino acid with a polar residue, which likely disrupts functionality [39]. Subsequent mutation detection efforts resulted in the identification of a second missense mutation (T 484 C transition; S 162 P missense mutation) in exon 5 of *Nle1* for the *l11Jus4* allele (Figure 1D). This non-conservative amino acid substitution has a high probability to alter protein function, as serines easily form hydrogen bonds with polar substrates, while prolines are rarely found in active sites [39]. In addition, we detected an endogenous C57BL/6J non-synonymous SNV (A 535 G transition; I 179 V) in exon 6 (Figure 1D). This well-documented SNV (rs2820949) leads to a very conservative amino acid substitution [39]. Both mutants share the endogenous mutation (Figure 1D, 1F), indicating that *l11Jus1* and *l11Jus4* homozygotes harbor two coding changes in *Nle1*–an ENU-induced allele and an endogenous C57BL/6J missense mutation.

### *Sequence variations within the* Nle1 *locus*

We identified 21 new polymorphisms and compiled a list of all of the polymorphisms found to date within the *Nle1* locus (MGI dbSNP Build 128) (Additional file 1: Table S1) [40]. *Nle1* is transcribed from the Crick strand, and variances are ordered in reference to *Nle1*, not the chromosome. The nucleotide position is noted in column one (NCBI Build 37) and the location within *Nle1* in column two. Variances detected within exons are designated as synonymous (S) or non-synonymous (N). Variances reported in the MGI database, but not found in our sequencing studies are depicted by gray cells, and dbSNP IDs for previously identified changes are noted.

We included known variants in the reference strain (C57BL/6J), as well as those found in the 129S1/SvImJ, C3H/HeJ, NOD and CzechII strains, and the strains that we sequenced (*l11Jus1*, *l11Jus4*, C57BL/6J, 129S6/SvEvTac, and C3HeB/FeJ). In total, there are 73 variances across the *Nle1* locus, including 5’ and 3’ UTR sequences. We saw no discordance between the C57BL/6J sequences and the reference sequence. We discovered 10 new indels; 129S6/SvEvTac and C3HeB/FeJ had identical sequences across these regions, while C57BL/6J, *l11Jus1* and *l11Jus4* segregated together. cDNA comparison studies also indicate that the NOD allele (Genbank Accession # AK170853.1) has a single nucleotide deletion in the 3’UTR sequence [41]. All polymorphisms identified in the 129S6/SvEvTac and C3HeB/FeJ strains are new. We identified five new expressed SNVs across the gene and one expressed indel in the 3’UTR. One previously reported expressed SNV (rs28209059) is a missense mutation (C 912 G; Additional file 1; Table S1) in the 129S1/SvImJ and C3J/HeJ strains. This results in a neutral amino acid substitution (N 291 K) [39] within exon 8, which does not encode for any type of functional domain.

### *The* Nle1 *locus*

**Figure 3:**
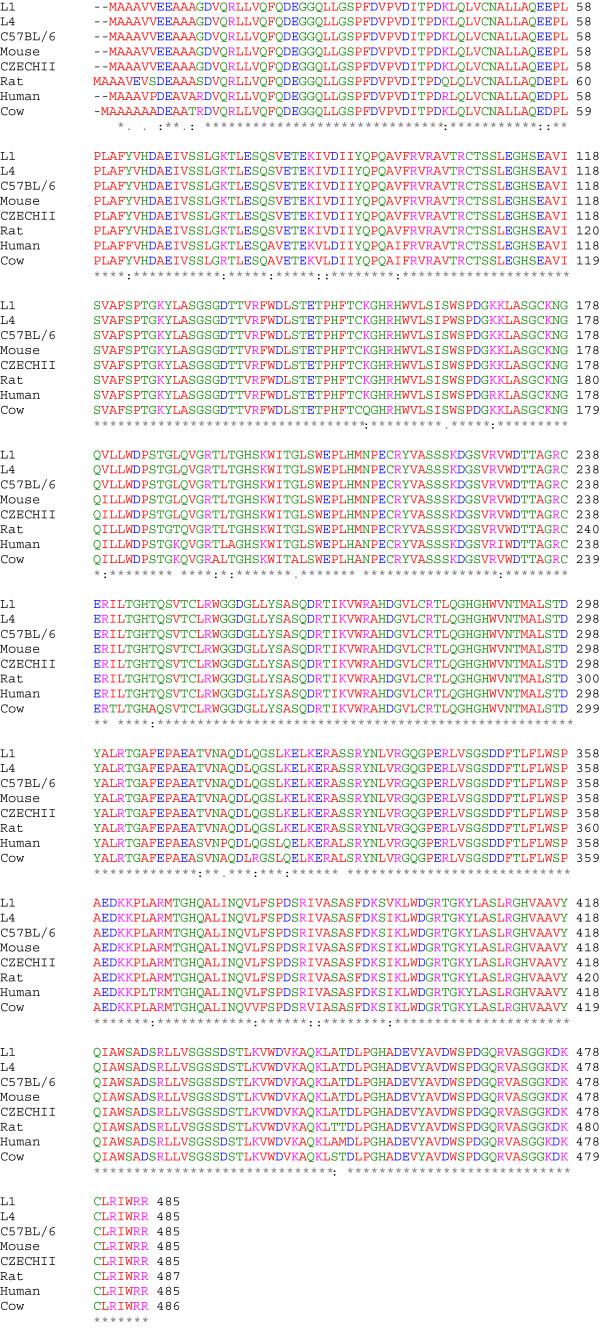
**NLE1 protein sequence alignment.** NLE1 is highly conserved among mouse, rat, cow and human.

BLAT analysis of the mouse RefSeq cDNA (Accession # NM_15431) at the UCSC genome browser [42], reveals that the *Nle1* locus contains 13 exons and spans 7628 bp of genomic DNA. cDNA and EST sequences indicate the potential for generating several alternatively-spliced transcripts. The RefSeq cDNA is predicted to encode a 485 amino acid protein that has an NLE1 domain at the N-terminus and 8 WD40-like repeats (Figure 1F). NLE1 is highly conserved, with orthologues in multiple species, even yeast and plants; it is over 91% identical among mouse, rat, human and cow (Figure 3). In addition, it is highly conserved in yeast (42%), fruit fly (55%) and potato (58%) (data not shown).

### *Expression analysis of the *Notch *pathway in mutant embryos*

Since *Nle1*^*l11Jus1/l11Jus1*^ embryos die shortly after implantation, while disruption of NOTCH signaling by multiple methods (i.e. targeted deletion of *Notch* receptors and ligands, [11-21], *γsecretase*[27] or *Pofut1*[25,26]) in mice leads to embryonic lethality after mid-gestation, we hypothesized that *Nle1*^*l11Jus1/l11Jus1*^ and *Nle1*^*l11Ju41/l11Jus4*^ mutants had defects in multiple signaling pathways. To test for defects in NOTCH signaling, we analyzed *Notch* pathway gene expression in homozygous mutant (i.e. *Nle1*^*l11Jus1/l11Jus1*^) blastocysts using the PAMM-059 *Notch* Pathway SAB PCR Array (SABiosciences/Qiagen, Frederick, MD). We compared expression levels of 84 *Notch* pathway genes in *Nle1*^*l11Jus1/l11Jus1*^ pre-implantation embryos to *Nle*^*+/+*^ control littermates.

We eliminated 19 genes, including 8 *Notch* downstream targets (*Cflar, Ifng, Il2ra, Pparγ, Cd44, Dtx1, Krt1 and Ptcra*) and the *Notch* ligand, *Dll1*, due to lack of expression (i.e. had a Ct value ≥ 29.5). This left 65 genes for statistical analysis, including the *Notch* receptors (*Notch1-4*), *Jagged* ligands (*Jag1*-*2*) and receptor processing and modifying enzymes (i.e. the γ-secretase complex and protein O-fucosyltransferase genes: *Adam10*, *Adam17*, *Psen1*, *Psen2, Psenen* and *Pofut1*).

*Notch* target genes include *Cdkn1a* (a marker of cell cycle arrest), *Hes1*, *Hey1, Stat6*, *Nr4a2, Nfkb1* and *Pparg* (transcriptional regulators), *Ccnd1* (cell cycle), as well as *Chuk*, *Il17b* and *Krt1* (downstream targets with unspecified functions in the NOTCH pathway). In addition, the PCR array contains several members of the *Wnt* (*Aes, Axin1, Lrp5, Fzd1-7* and *Wnt11*) and Hedgehog signaling pathways (*Gli1, Gsk3b, Shh, Smo* and *Sufu*). To ensure biological significance, genes with less than a 1.5 fold change were included in the statistical analyses, but not considered differentially expressed.

We analyzed the data using a GLM blocked by plate (technical replicate) nested within pool (biological replicate) and pool nested within genotype. Transcript levels were nested within biological function/pathway (role). Role and transcript were crossed with the blocking factors plate and pool, and with the experimental factor genotype. Therefore, testing for genotype X transcript interactions will identify single genes that are statistically upregulated or downregulated in mutant embryos, while testing for genotype X role interactions identifies groups of genes with similar biological functions (i.e. *Wnt* pathway, transcriptional regulation, etc.) that are as a whole misregulated in mutant embryos compared to wild-type controls. The role (GLM: F_7,84_ = 248.3; P < 0.0001) and transcript (F_7,84_ = 224.0; P < 0.0001) effects were significant, indicating the presence of consistent functional and expression profiles in both genotypes.

**Figure 4:**
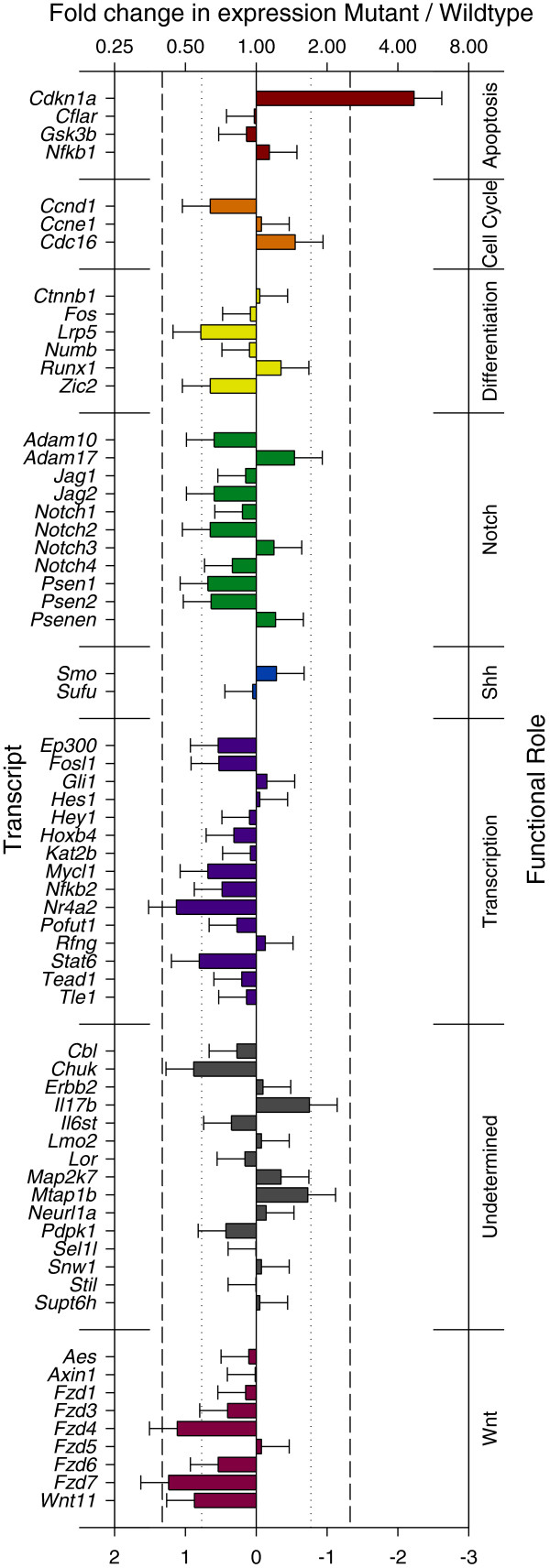
**Expression of *Nle1***^***l11Jus1 ***^**embryos using a*Notch*-specific PCR array.** Each transcript is listed (left), with fold change indicated (top). The mean expression level for each transcript is indicated. The least-squares mean ΔΔCT ± SE is shown for each transcript, with equivalent fold-change. The dashed line indicates that *Cdkn1a* is the only gene with significant differences in expression following a Bonferonni correction for multiple comparisons. The dotted line represents transcripts where p < 0.05. Biological roles were taken directly from the SAB website.

The genotype X transcript interaction was significant (F_57,684_ = 1.5490; P = 0.0073), indicating that at least one transcript differed from the overall mean of transcripts within the same role. Of the 16 *Notch* target genes detected in this study, 6 were overexpressed by at least 1.5 fold (*Cdkn1a, Nfkb1, Hes1, Erbb2 (Esr2), Il17b, Map2k7*). However only *Cdkn1a*, which was upregulated by 4.7 fold in mutant samples (Figure 4) (p = 1.94X10^-8^), was statistically significant using *post hoc* tests corrected for multiple comparisons (accepting p < 0.000769); none of the other genes approached significance, even at an uncorrected threshold of p < 0.05.

Seven genes demonstrated a more than 1.5 fold reduction in expression in *Nle1*^*l11Jus1/l11Jus1*^ embryos: *Lrp5, Fzd7, Fzd4* and *Wnt11,* which are members of the *Wnt* signaling pathway, and *Chuk*, *Nr4a2* and *Stat6*. However, none of these met the rigorous criteria (p < 0.000769) that accounts for the multiple comparison analysis. Setting aside Bonferroni corrections for false positive detection rate, and accepting a false discovery rate (FDR) of 5% leads to the inclusion of *Fzd7* (p = 0.00168), *Nr4a2* (p = 0.00422), *Fzd4* (p = 0.00461) and *Chuk* (p = 0.02492); using an FDR of 10% leads to the inclusion of *Wnt11* (p = 0.02679) (Figure 4). This analysis indicates that multiple members of the *Wnt* pathway are downregulated in *Nle1* mutant embryos.

### *Confirmation of* Cdkn1a *in different stages of pre-implantation development*

The SAB PCR array study was performed on three pools (n = 15) of E3.5 embryos at different stages (i.e. a mix of morula, blastocysts and hatched blastocysts). This could have introduced biased expression or masked subtle alterations in stage-specific gene expression due to pooling of embryos from different stages. To control for these possibilities and confirm the PCR array results, we analyzed expression of *Cdkn1a* between wild-type and mutant embryos at multiple embryonic stages (morula, full blastocyst and hatched blastocyst) in single embryos using TaqMan assays. Expression of each gene was normalized relative to expression of *Gapdh* and compared to the stage-matched wild-type controls. Wild-type expression was set at a value of one.

We used a multivariate GLM model to calculate ΔΔCt and fold change and to properly control and test for differences between the mutant alleles and stages of development. Using a least squares mean, which corrected for all of the variables in the analysis (line, genotype, stage of development), we did not detect differences in *Cdkn1a* expression between mutants compared to control embryos as a function of stage (morula, blastocyst and hatched blastocyst; F_2,32_ = 0.2701; P = 0.7650), line (*Nle1*^*l11Jus1*^ and *Nle1*^*l11Jus4*^; F_1,32_ = 1.293; P = 0.2640) or stage and line (F_2,32_ = 0.0574; P = 0.9444), indicating that we did not detect differences in expression due to developmental stage or mutant line. However, overall*, Cdkn1a* was expressed at 4.69 fold (95% CI: 1.02 - 21.5 fold) higher levels both in *Nle1*^*l11Jus1/l11Jus1*^ and *Nle1*^*l11Jus4/l11Jus4*^ mutant embryos compared to wild-type controls at all stages (GLM: F_1,32_ = 4.2561; P = 0.0473). These data are consistent with our PCR array findings for *l11Jus1* mutant embryos, show that there are no significant differences in expression of *Cdkn1a* at the different stages tested in the PCR array, and demonstrate that the phenotypes associated with *l11Jus1* and *l11Jus4* do not differ at the molecular level.

### *Apoptosis occurs at E4.5* in Nle1^l11Jus1/l11Jus1^*and* Nle1^l11Jus4/l11Jus4^*mutants*

These *Cdkn1a* expression findings are intriguing, as targeted disruption of *Nle1* in mice indicated that mutant embryos started to undergo apoptosis at E3.5 plus 1 day in culture, which is approximately equivalent to E4.5 embryos (i.e. hatched blastocysts) [30]. If our mutant embryos were also undergoing apoptosis, we would expect that they would not show high levels of *Cdkn1a*, as *Cdkn1a* expression is most often an indication that cells have exited the cell cycle following a DNA damage response or other type of cellular stress event [43,44]. However the function of CDKN1A in cell cycle arrest and apoptosis is still unclear, as other studies have shown that *Cdkn1a* expression levels can be upregulated in cells undergoing apoptosis [45]. Given that *Cdkn1a* was expressed at much higher levels in *Nle1* mutants compared to wild-type embryos, we hypothesized that animals expressing high levels of *Cdkn1a* would be protected from apoptosis at E3.5, but would ultimately become apoptotic by E4.5, which would be consistent with previous studies [30].

**Figure 5:**
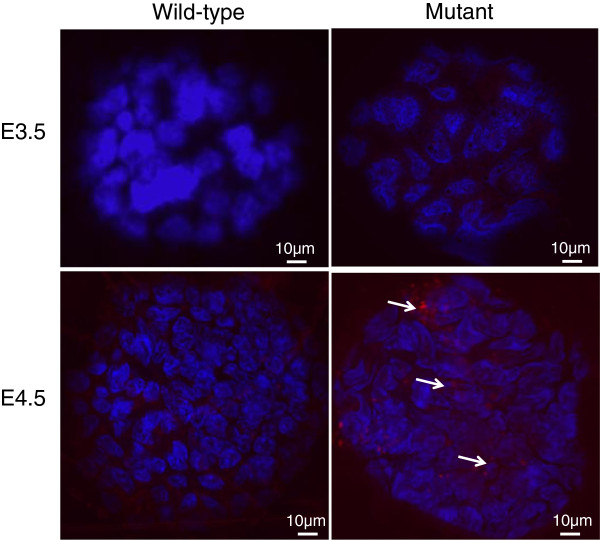
**Caspase 3 detection in *Nle1***^***l11Jus1 ***^**mutant embryos.** caspase 3 detection in mutant and wild type embryos at E3.5 (200X) and E4.5 (200X). Positive caspase 3 staining is only detected in E4.5 mutants. Red staining indicates the presence of caspase 3 signal and blue is Hoechst staining. White arrows point out caspase staining. Merged images are shown.

Therefore, we analyzed caspase 3 activity at E3.5 and E4.5 (Figure 5; Table 4). We tested these data in a single logistic multiple regression. We detected caspase 3 activity solely in homozygous mutants (Likelihood Ratio *χ*^2^ = 21.75, p < 0.0001) at E4.5 (LR *χ*^2^ = 15.11; p < 0.0001). Strain had no effect (LR *χ*^2^ < 0.0001; p > 0.9999). We did not find any evidence for an interaction between genotype and stage (LR *χ*^2^ < 0.0001; p > 0.9999). Overall, these data indicate that apoptosis appears at E4.5, but not at E3.5, that apoptosis is only seen in homozygous mutants and that these two effects are independent and additive in both alleles.

**Table 4:** Logistic multiple regression analysis of caspase 3 detection

**Genotype/stage**	**129/129**		**Mutant/129**		**Mutant/Mutant**		**Total**
**caspase 3**	**+**	**–**	**+**	**–**	**+**	**–**	
*l11Jus1* E3.5 morula/ blastocyst	0	1	0	5	0	2	8
*l11Jus1* E4.5 hatched blastocyst	0	2	0	5	2	0	9
*l11Jus4* E3.5 morula/ blastocyst	0	4	0	5	0	3	12
*l11Jus4* E4.5 hatched blastocyst	0	3	0	4	3	0	10

### *qRT-PCR analysis of* Trp53 *in mutant embryos*

We then asked whether the apoptotic phenotype is *Trp53*-dependent. Taqman assays were conducted to compare *Trp53* expression between wild-type and mutant embryos. Identical GLM models were used to calculate fold change and test significance, with additional planned contrasts performed to test differences at different embryonic stages. *Trp53* expression did not differ between control and mutant embryos overall (GLM: F_1,33_ = 0.5316; P = 0.4711), by line (F_1,33_ = 0.0057; P = 0.9404), by embryonic stage (F_2,33_ = 0.0994; P = 0.9056), or the interaction of line and stage (F_2,33_ = 0.1782; P = 0.8376).

Together, the gene expression and caspase 3 data indicate that *Nle1*^*l11Jus1/l11Jus1*^ and *Nle1*^*l11Jus4/l11Jus4*^ homozygotes undergo caspase 3-mediated apoptosis at the hatched blastocyst stage. This is not mediated by alterations in mRNA levels of *Trp53*, and apoptosis does not correlate with upregulation of *Cdkn1a* expression in homozygous mutant embryos, as upregulation of *Cdnk1a* occurs from the morula through the hatched blastocyst stage, even though apoptosis only is observed at the latest stages. Furthermore, we demonstrate that several members of the *Wnt* pathway are downregulated in mutant embryos, suggesting that NLE1 interacts with the WNT pathway during pre-implantation development.

## Discussion

We present evidence that the *l11Jus1* and *l11Jus4* mutant phenotypes are caused by non-conservative missense mutations in the *Nle1* gene. Gene targeting studies indicate that *l11Jus1* and *l11Jus4* phenocopy the null allele [5]. These ENU mutants were created on a C57BL/6J background, which also contains a conservative missense mutation in *Nle1*. Therefore, *l11Jus1* and *l11Jus4* homozygotes harbor two mutations within predicted functional domains of NLE1. Previous studies in *Drosophila* and *Xenopus* indicate that NLE1 is a member of the NOTCH pathway [5,6]. NOTCH signaling facilitates short-range cell-cell communication during diverse cellular processes, in multiple tissues and at a multitude of developmental stages. Loss of function or gain of function mutations in various factors that are fundamental to canonical NOTCH signaling are often associated with developmental disorders, adult-onset diseases and a variety of cancers in humans [22].

However, the function of NLE1 in NOTCH signaling remains elusive. Initial studies in *Drosophila* show that NLE1 function is context and dosage dependent. NLE1 was identified as a dominant suppressor of the viable mutant allele, *notchoid*[6]. *Notchoid* mutants have characteristic wing notches; in the *notchoid* mutant background, *Nle1* heterozygosity (i.e. *Nle1*/+) rescued the *notchoid* phenotype, while simultaneously causing shortened and thickened wing veins [6]. Interestingly, overexpression of wingless, the *Drosophila Wnt* orthologue, in the *notchoid* background also rescues the *notchoid* phenotype [46,47], while overexpression of *Notch* leads to shortened, thickened wing veins [48]. Since NLE1 can bind the NOTCH intracellular domain (NICD), these experiments suggest that NLE1 is a negative regulator of NOTCH, and that NLE1 functions by blocking the ability of the NICD to regulate expression of downstream targets. Studies in *Xenopus* come to the opposite conclusion, as overexpression of *Murine*[5] or a combination of *Xenopus* and *Drosophila*[6]*Nle1* mRNAs into single blastomeres at the 4-cell or 2-cell stage, respectively, lead to decreased numbers of primary neurons at the early neurula stage. These results indicate that NOTCH activity was upregulated following injection of *Nle1* mRNAs, suggesting that NLE1 positively regulates NOTCH signaling.

These studies indicate that NLE1 acts as both a positive and negative regulator of NOTCH signaling. If NLE1 acts as a general positive regulator of NOTCH signaling during murine pre-implantation development, then elimination of NLE1 could lead to compensatory over-expression of the *Notch* receptors, ligands and protease family members, but reduced expression of downstream target genes. In contrast, if NLE1 functions as a negative regulator of NOTCH signaling, we would predict that disruption of NLE1 would lead to increased expression of *Notch* target genes. To our surprise, we saw no generalized misregulation of *Notch* target genes in the PCR array study. In addition, the *Notch* receptors, ligands and other family members were not significantly altered. At a false discovery rate of 5%, the only genes that are misregulated in the PCR array study were: *Cdkn1a*, *Nr4a2*, *Fzd7*, *Fzd4* and *Chuk*. *Cdkn1a*, *Nr4a2* and *Chuk* are downstream targets of NOTCH signaling, while *Fzd7* and *Fzd4* encode receptors in the WNT pathway. To tease out the gene expression changes that occurred during specific pre-implantation stages, we analyzed expression of *Cdkn1a* on individual staged embryos (morulae, full blastocysts and hatched blastocysts). *Cdkn1a* was significantly upregulated in *Nle1*^*l11Jus1/l11Jus1*^*and Nle1*^*l11Jus4/l11Jus4*^ animals at all three stages. These studies indicate that mutations in *Nle1* do not significantly affect the NOTCH pathway during pre-implantation development.

CDKN1A is a powerful cyclin-dependent kinase inhibitor that functions in several developmental pathways and negatively regulates the cell cycle at G_1_ via a TRP53-mediated response to DNA damage [49]. This can happen directly by competing with DNA polymerase δ for PCNA binding sites at the replication fork, leading to decreased DNA synthesis [50]. Alternatively, CDKN1A can inhibit CDK2, which leads to suppression of E2F-dependent transcripts, downregulation of components of the DNA synthesis machinery and reduced firing at origins of replication [51]. In addition, CDKN1A can act as a negative regulator of caspase-mediated apoptosis [49]. Gene targeting studies demonstrated that the inner cell mass of *Nle1*^*−/−*^ embryos was undergoing apoptosis via a caspase-dependent mechanism in E3.5 blastocysts that were cultured for 24 hours [5]. We analyzed caspase 3 activity in blastocysts and hatched blastocysts at E3.5 and E4.5. Consistent with the results of Cormier and colleagues (2006), we demonstrate that *l11Jus1* and *l11Jus4* show evidence of apoptosis only in hatched blastocysts.

Although we show upregulation of *Cdkn1a* and downregulation of several members of the *Wnt* pathway, how these two networks work together to regulate pre-implantation development is still unknown. One attractive possibility is via the TRP53-mediated stress response pathway, which is upstream of CDKN1A. We predicted that if our *Nle1* mutations were causing severe cellular damage, the cell would not be able to recover during cell cycle arrest (at E3.5), which would then force the cell to undergo apoptosis (at E4.5). If this were true, we would expect to see increased expression of *Trp53*, as the cells proceeded through apoptosis. However, we did not detect altered expression of *Trp53* by qRT-PCR studies in our mutants at any stage. In retrospect, this result is not that surprising, as TRP53-mediated apoptosis (via *Cdkn1a* upregulation) is not necessarily correlated with mRNA expression of *Trp53*, but is instead associated with upregulation of the active, acetylated form of the TRP53 protein [52]. Alternately, it is possible that the expression changes in *Trp53* were too subtle to detect, or apoptosis could be occurring via non-TRP53 mediated pathways [53].

## Conclusions

Our results refute the possibility that NLE1 functions as a negative regulator of NOTCH signaling during mammalian pre-implantation development, as mutation of *Nle1* does not lead to increased expression of key *Notch* downstream target genes. In addition, our PCR array studies indicate that most downstream targets of NOTCH are unaffected by *Nle1* mutations. Much to our surprise, instead of confirming the role of NLE1 in the NOTCH pathway, our data implicate NLE1 in WNT signaling and cell cycle arrest and/or apoptosis via *Cdkn1a* during pre-implantation development. Although NOTCH signaling is dispensable in mice prior to gastrulation, WNT signaling is not. Deletion of *Wnt3* leads to failure prior to primitive streak formation [54], and multiple *Wnt* ligands and *Fzd* receptors are detected at the blastocyst stage [55,56], as well as in the uterus during peri-implantation [57-59]. We provide evidence that NLE1 is co-opted by the WNT and CDKN1A pathways in mammals, while in lower vertebrates, several studies indicate that NLE1 acts in the NOTCH pathway, and reports in yeast and plants (which lack NOTCH signaling) demonstrate a role for NLE1 in ribosomal biogenesis [60-64]. Therefore, NLE1 may perform widely varied functions in a species and stage-dependent context, and out studies suggest that NLE1 may co-opt different signaling pathways during different stages of development.

## Abbreviations

GLM: General linear model; Mb: Megabase; RNA: Ribonucleic acid; DNA: Deoxyribonucleic acid; cDNA: Complementary deoxyribonucleic acid; EST: Expressed sequence tag; qRT-PCR: Quantitative reverse transcription PCR; PFA: Paraformaldehyde; Nle1: Notchless homologue 1 (*Drosophila*); Trp53: Transformation related protein 53; Cdkn1a: Cyclin dependent kinase inhibitor 1a (p21); BLAT: BLAST like alignment tool; Ct: Threshold cycle; the relative measure of the target in a quantitative PCR reaction.

## Competing interests

The authors acknowledge no competing interests.

## Authors’ contributions

ACL conceived of and participated in the design of the study; conducted the meiotic mapping and haplotype studies; identified the critical interval; supervised C-LL, KMB and MJC; and drafted the manuscript. C-LL participated in the design of the study, conducted the qRT-PCR studies and caspase 3 analysis and drafted corresponding parts of the manuscript. KMB participated in the design of the study, identified the *Nle1*^*l11Jus4*^ mutation, conducted the PCR array study and drafted corresponding parts of the manuscript. MJC participated in the design of the study, found the *Nle1*^*l11Jus1*^ mutation and edited the manuscript. JPG conducted the statistical analysis for the SAB PCR arrays, TaqMan assays and caspase 3 studies and edited the manuscript. MJJ conceived of the study, participated in the design and edited the manuscript. All authors read and approved the final manuscript.

## Authors’ informations

ACL initiated this project while a postdoctoral fellow under the supervision of MJJ at Baylor College of Medicine. All gene expression studies were conducted under the direction of ACL at Purdue University.

## Supplementary Material

Additional file 1 **Table S1.** Polymorphisms in the Nle1 locus. Click here for file
